# Clinical Instruction

**Published:** 1847-11

**Authors:** 


### EDITORIAL DEPARTMENT.

*Clinical Instruction.*—The claims of clinical instruction are now so well understood and generally appreciated by the medical profession, that to propose it as a topic for discussion in the pages of a medical journal, would be deemed ridiculous, and to amplify upon its importance, a work of supererogation. We have no intention of insulting the intelligence of our medical readers by a formal advocacy of the advantages which accrue to medical science, and to the public, by associating, as far as practicable, with didactic, bed-side teaching. By the former the prominent facts of medical science may be presented, its principles inculcated, its methods of investigation explained, and, in a word, its philosophy fully expounded; but by the latter, pursued conjunctively with the former, these facts are demonstrated, principles illustrated, methodical investigations exhibited, and the philosophy, in a measure, brought under the cognizance of the senses. In view of the great importance of clinical instruction (which has been more and more appreciated as our science has advanced, and greater attention bestowed on the subject of medical education;) efforts have been made, and are now being made to incorporate it more fully with other branches of the study of medicine, and, at the same time, to develop and increase the availableness of its resources. As we have said, we need not occupy space, at this day, to convince medical men of its value as an element of medical education, and we have no design to intrude considerations which are familiar even to triteness; but it has occurred to us, that a few remarks respecting the means by which objects of clinical instruction are to be secured, and the degree of responsibility for the furtherance of these objects which rests upon the profession at large, may not be regarded as entirely misplaced, or superfluous.

There are two sources whence facilities for clinical instruction are derived. One of these is Dispensary practice, and public charitable institutions, like alms houses and hospitals, constitute the other. The first is excellent as far as it goes. It is abundantly available for a large proportion of surgical, and a considerable number of medical diseases. The establishment of dispensaries, or *cliniques* as they are miscalled, in most of our medical schools, is a plan of somewhat recent date, and in our view, is productive of much good; but it is obvious that it excludes diseases, and injuries which confine patients to the bed. So far as the latter are concerned, clinical, or bed-side instruction, in the literal signification of these terms, is alone applicable, and this can only be given at the public institutions named. In France, England, Germany, and we suppose, most countries of civilized Europe, these institutions are made subservient to purposes of medical education. The medical officers are generally teachers, and sometimes it is rendered obligatory that the physicians and surgeons connected with such institutions shall devote a certain portion of their time to oral instruction. It was one of the most prominent of the recommendations adopted by the late medical convention in France, that this obligation shall be enforced in all cases. In the report of the committee on medical education adopted by the second American National Medical Convention, this subject was urged upon the attention of the profession. But no authority, nor argument need be adduced to sustain the broad principle, that science, medical education, and public welfare, are alike promoted by making all charities instituted for the support of persons diseased or disabled, *Schools of Medicine.* No intelligent medical man, surely, will refuse his assent to this principle. But, with respect to this,as as well many other subjects connected with the medical profession, the public requires to be informed, and the objects of clinical instruction cannot be fully secured, until this end is accomplished; for many, if not most charities are not only dependent upon the public for their support, but public sentiment controls the details of their management. Who are to enlighten the public on this subject, to explain its importance, to show its direct bearings upon the best interests of the community, and to remove prejudices which may exist, if not the medical profession? How can the end be attained except the profession be as united in action and influence, as they are really in opinion and sentiment upon this subject? If these interrogatories convey the force which they appear to us to possess, it must be apparent that the medical profession is in fact responsible for the co-operation of the public in the matter of which we are speaking. The sensible portion of every community cannot fail to adopt just and liberal views respecting it, if it be fairly presented; they will realize its importance, and see the extent to which their own interests are involved in the action which they may take respecting it. Here, as in the instance of the legalization of Anatomy, all that is wanting is for the profession to be true to itself, and to the requisitions of science and humanity. The profession have it in their power to so enlighten popular sentiment on the subject, that it will never consent that medical education shall be deprived of the advantages of clinical instruction. It would be a libel on the profession to assert that the united efforts of its members are insufficient to place this, as well as other kindred subjects, before the public in such a light, that it will cheerfully yield its co-operation in measures tending to promote and extend the usefulness of medicine. We would respectfully, but earnestly commend this train of reflection to our readers, and ask each to inquire of himself, in how far am I personally chargeable with the indifference, or indisposition on the part of the public to provide facilities for clinical instruction, by consenting, nay, insisting that charitable institutions shall be made subservient to that object.

In facilities for clinical instruction, medical education labors under embarrassments in this country, greater than in France or Great Britain. If we mistake not, the superior educational advantages of some European schools over those of our own country, consist chiefly, or entirely, in their having at control a greater extent of these facilities. In conjunction with these facilities, in England and France, more particularly in the latter, are more ample opportunities for the various pathological researches to which public institutions are eminently adapted; and hence it is that the profession of our country is not furnishing its quota to the accumulating stock of medical knowledge. We believe we do not assume too much in saying, that the medical profession of the United States embraces a larger amount of talent and industry than would perhaps appear from a comparison with some other countries as regards results conducive to the progress of medical science; and one reason, if not the chief reason for this contrast in such results is, that our Hospitals and Alms-houses are less extensive in the first place, but, in the second place, they are less available for scientific purposes. Why is it that from Paris have emanated so many valuable discoveries and improvements in all the departments of medicine and surgery, and that such astonishing zeal pervades the entire profession? The answer to this question, is, that the Public Eleemosynary institutions of Paris are tributary to medical education and the advancement of medical science, as well as to the more immediate ends of charitable relief. These objects, instead of conflicting with each other, ensure a greater mutual advantage. Scientific interest increases the direct benefits which are derived by those who become candidates for public charities; the inducements furnished by the responsibilities of teaching, develop greater ardor of investigation, and the latter obviously contributes to the value of the instructions given. Thus, each separate branch of utility, the more effectually it is carried out, provides for the more complete fulfilment of the other branches. These remarks express mere truisms, which every reflecting Physician considers as such. But it is not enough that they are so regarded by the Profession. The public should so regard them, and it is for members of the medical profession, in their intercourse with the community around them, and by such associated action as shall seem judicious, to place the subject in a proper light before the public, so as to secure, in behalf of medical education, and medical science, in this country, facilities not inferior to those elsewhere enjoyed.

Since the foregoing was penned, we find some evidences that the Profession and Public are becoming more alive to the subject in the city of New-York, by an editorial article in the Annalist. The extensive Alms-house Hospital at Bellevue, it seems, has not heretofore been conducted with reference to clinical instruction; but a change is about to be made, in which this and other scientific objects are to receive their proper share of attention. We will take the liberty of quoting a few paragraphs from the article referred to, containing views and wishes in which every sincere friend of our science and profession will heartily concur:—

"It seems now to be generally agreed, that the report of the Committee of the Common Council, charged with the reform in the Medical Department of this Institution, will be accepted by the City Fathers. This important measure has long been ardently demanded by the Profession, and they, to whose good sense and philantrophy it shall owe its adoption, will richly deserve its thanks. * * * It has long been a disgrace and an injury to American Science, that an institution, embracing such an infinity of cases in every department of practice, offering so extensive a field for the exercise of the skill and sagacity of many members of the healing art, and so valuable a school for its junior aspirants, should have contributed nothing to the advancement of medical learning, and have been closed against those who would have done honor to its name, and whose zeal would have advantaged its suffering inmates; the recessess of which would have opened to the student an exhaustless store of practical and pathological information.

We hope, ere long, to see the Bellevue Hospital, officered by an efficient medical and surgical staff, take its proper rank among the scientific and humane institutions of our land; contributing, as it ought, to the advancement of our noble science and the glory of American Medicine. We hope to see these medical officers earning for themselves an immortal reputation, and not less alleviating by their skill the sufferings of those entrusted to their care, than fitting themselves for the successful exercise of their professional duties, by the knowledge they there acquire; communicating to the world, to their own honor and the good of the Medical Art and of humanity, the ample and rich results of their observation and experience; followed by a crowd of admiring and attentive students, who, from their lips, may learn to pursue the path which has led them to eminence, and to uphold after them the dignity and the usefulness of their art. It has ever been our most ardent wish to see the Hospitals of America rank proudly, in scientific fame and in public utility, with the most celebrated of Europe; and for the attainment of this end, our most fervent aspirations will be unceasingly offered.

